# LONGITUDINAL CHANGES IN PROTEOMIC BIOMARKERS IN ALZHEIMER’S DISEASE AND CARDIOVASCULAR DISEASE

**DOI:** 10.1093/geroni/igad104.3715

**Published:** 2023-12-21

**Authors:** Laurie Theeke, Ying Liu, Silas Wang, R Osvaldo Navia, Danqing Xiao, Chun Xu, Kesheng Wang

**Affiliations:** The George Washington University, Washington, District of Columbia, United States; East Tennessee State University, Johnson City, Tennessee, United States; Carnegie Mellon University, Pittsburgh, Pennsylvania, United States; West Virginia University, Morgantown, West Virginia, United States; Regis College, Weston, Massachusetts, United States; University of Texas Rio Grande Valley, Brownsville, Texas, United States; School of Nursing, Health Sciences Center, West Virginia University, Morgantown, West Virginia, United States

## Abstract

Alzheimer’s disease (AD) and cardiovascular diseases (CVDs) commonly co-occur in older adults. This correlation prompts an investigation into the potential shared risk factors linking AD and CVDs, and the potential direct causative role of cardiovascular risk factors in AD. The cohort comprised 566 older adults including 111 individuals with AD, 58 without AD, 383 with mild cognitive impairment (MCI), 410 with CVD and 156 without CVD from the Alzheimer’s Disease Neuroimaging Initiative (ADNI). The multivariable linear mixed model (LMM) was used to investigate the associations of AD and CVD on longitudinal changes of 146 proteomic biomarkers (measured at baseline and 12-month follow-up), while adjusted for age, gender, education, racial groups, and APOE-ε4 alleles. The LMM showed that AD and CVD were associated with longitudinal changes in 48 and 46 proteomic biomarkers, respectively (p< 0.05). Interestingly, both AD and CVD were associated with longitudinal changes in 14 proteomic biomarkers (A1Micro, ApoH, B2M, BNP, Complement C3, Cystatin C, KIM1, NGAL, PPP, TIM1, THP, TFF3, TM, and VEGF). Furthermore, the single biggest risk gene for sporadic AD, APOE-ε4 was associated with 22 proteomic biomarkers. Moreover, MCI and CVD were associated with 13 proteomic biomarkers (A1Micro, ApoD, AXL, BNP, Calcitonin, CD40, C-peptide, pM, PPP, THP, TNFR2, TTR, and VEGF), while MCI, CVD and APOE-ε4 were associated with four proteomic biomarkers (A1Micro, Calcitonin, THP, and TNFR2). This study provides valuable insights into the shared and distinct pathophysiological mechanisms at play, offering a stepping-stone towards a deeper comprehension of the interrelationship between AD and CVDs.

